# Bactericidal Efficacy of Sodium Hypochlorite on Eggshells Contaminated with Three *Salmonella* Serovars

**DOI:** 10.3390/pathogens15020133

**Published:** 2026-01-26

**Authors:** Min-Ho Park, Seok-Jin Cho, Kyoung-Seong Choi

**Affiliations:** 1 Department of Animal Science and Biotechnology, College of Ecology and Environmental Science, Kyungpook National University, Sangju 37224, Republic of Korea; sksalsgh11@naver.com; 2 Department of Ecological Science, College of Ecology and Environmental Science, Kyungpook National University, Sangju 37224, Republic of Korea; sjcho001002@naver.com

**Keywords:** *Salmonella* reduction, eggshell sanitation, washing temperature, exposure time, food safety

## Abstract

Eggs are nutrient-dense yet recognized vehicles for *Salmonella* transmission. Because eggshells are easily contaminated during production and handling, washing is critical to reduce microbial load. Here, we evaluated the bactericidal efficacy of 150 ppm sodium hypochlorite (NaOCl) against *Salmonella enterica* subsp. *enterica* serovar Enteritidis (*S.* Enteritidis), serovar Typhimurium (*S.* Typhimurium), and serovar Thompson (*S.* Thompson) across washing temperatures and exposure times. Eggs were inoculated by immersion in 100 mL of a *Salmonella* suspensions (approximately 8 log CFU/mL) prepared separately for each serovar, air-dried for 1 h, and subsequently washed in 150 ppm NaOCl for 35, 40, 45, or 50 °C. Following washing, eggs were transferred to buffered peptone water, homogenized, and plated on xylose lysine deoxycholate agar to quantify the residual *Salmonella* populations on the eggshells. Washing with 150 ppm NaOCl significantly reduced counts (3–5 log) under all conditions versus unwashed eggs (*p* < 0.001). Maximum inactivation for *S.* Enteritidis occurred at 35 °C for 45 s (4.95 log), whereas *S.* Typhimurium was greatest at 45 °C for 45 s (5.48 log). In contrast, *S.* Thompson showed a nonmonotonic, time-dependent pattern, with maximum inactivation occurring at 40 °C for 45 s (5.17 log). Overall, 150 ppm NaOCl effectively reduced *S.* Enteritidis, *S.* Typhimurium, and *S.* Thompson on eggshells. Efficacy appeared to be serovar dependent, with maximal reduction occurring at different temperatures for each serovar. These findings support standardized egg-washing guidelines to minimize *Salmonella* transmission across the food supply chain.

## 1. Introduction

*Salmonella* species are leading pathogens of foodborne illness worldwide, causing acute gastroenteritis, bacteremia, and meningitis [1]. Non-typhoidal salmonellosis causes an estimated 95 million foodborne infections and 50,771 deaths annually and is an emerging global public health issue [2]. Numerous cases and outbreaks have been linked to the consumption of eggs and egg products worldwide [3,4,5,6]. Eggs are widely consumed, nutrient-dense foods, yet their association with foodborne pathogens—particularly *Salmonella*—poses a major public health concern [7]. Moreover, eggs can be contaminated via vertical or horizontal transmission during formation in the layer hen or during handling along the supply chain [8]. In the Republic of Korea, *Salmonella* caused 204 foodborne outbreaks and approximately 7788 cases reported during 2020–2024 [9]. Notably, eggs and egg-derived products accounted for about 76–77% of salmonellosis cases [5,10].

Among more than 2600 *Salmonella* serovars, *Salmonella enterica* subsp. *enterica* serovar Enteritidis (*S.* Enteritidis), serovar Typhimurium (*S.* Typhimurium), and serovar Thompson (*S.* Thompson) are the most common causes of foodborne infection and are highly relevant to public health [11]. Recently, *S.* Thompson has emerged as a serovar associated with foodborne outbreaks. In the Republic of Korea, a large gastroenteritis outbreak (*n* = 2207) was traced to egg whites used to prepare a chocolate cake [12]. Accordingly, in 2019, the Republic of Korea revised microbial inspection standards for edible eggs to require the absence of *S.* Enteritidis, *S.* Typhimurium, and *S.* Thompson in edible eggs [13]. Including these three serovars is therefore pertinent to egg-related salmonellosis epidemiology and evaluation of sanitation strategies.

Egg washing reduces eggshell microbial loads and may decrease foodborne illness [14,15]. Egg washing is prohibited in the European Union (EU), whereas the United States, Japan, and Australia employ washing—often with 100–200 ppm sodium hypochlorite (NaOCl) within food safety management systems [5]. In poultry production, microbial control commonly relies on chlorine-based disinfectants such as NaOCl and chlorine dioxide (ClO_2_), which are valued for ease of use, antimicrobial activity, established safety for food-contact applications, and cost-effectiveness [16,17]. NaOCl is widely used in the food industry because it disrupts bacterial cell walls and denatures proteins [18]. Effectiveness nevertheless depends on pH, temperature, concentration, contact time, surface properties, and organic load. In the Republic of Korea, the Ministry of Food and Drug Safety (MFDS) approves NaOCl as a food-grade sanitizer for food, utensils, containers, and packaging. However, compared to other countries that use standardized protocols, the Republic of Korea has not yet established specific and unified guidelines for egg washing. Consequently, there is an urgent need to develop optimized washing standards to ensure egg safety and quality in the domestic market.

Therefore, to provide a scientific basis for establishing egg-washing guidelines in the Republic of Korea, we evaluated the bactericidal efficacy of 150 ppm NaOCl—a concentration within the internationally used range of 100–200 ppm—against *Salmonella* Enteritidis, Typhimurium, and Thompson across various washing temperatures and exposure times.

## 2. Materials and Methods

### 2.1. Egg Preparation

Fresh eggs were collected from a local farm housing brown laying hens in cages (33–53 weeks old). Eggs were refrigerated immediately after collection and equilibrated to room temperature before testing. Each egg was sanitized with 70% ethanol to remove surface bacteria and air-dried for 15 min in a biosafety cabinet. Each egg was then homogenized with 45 mL of 0.1% buffered peptone water (BPW; BNF Korea Co., Ltd., Gimpo, Republic of Korea) on a rocker for 2 min. Aliquots (100 μL) of the homogenate were plated on tryptic soy agar (TSA; BNF Korea Co., Ltd.) and incubated at 37 °C for 24 h. No bacterial growth was detected on TSA (BNF Korea Co., Ltd.) plates, confirming the absence of culturable bacteria on the egg surface and eggs were subsequently used in experiments.

### 2.2. Bacterial Strains and Egg Inoculation

Three *Salmonella enterica* serovars (*Salmonella* Enteritidis NCCP No. 14392, *S.* Typhimurium NCCP No. 13578, and *S.* Thompson NCCP No. 11718) were obtained from the National Culture Collection for Pathogens (Cheongju, Republic of Korea). Bacterial cultures were prepared according to the Korean Food Code (MFDS), as described previously [19]. Each stock was suspended in 1 mL of BPW (BNF Korea Co., Ltd.). For enrichment, a 100 μL aliquot was inoculated into 10 mL of BPW (BNF Korea Co., Ltd.) and incubated at 37 °C for 24 h. Subsequently, the cultures were subcultured into 500 mL of BPW (BNF Korea Co., Ltd.) and incubated at 37 °C for 24 h in a shaking incubator at 150 rpm. The optical density (OD_600_) was measured using 1 mL aliquots, and bacterial suspensions were adjusted to the target CFU for each strain. After enumeration, eggs (*n* = 4 per group) were individually immersed in 100 mL of the respective *Salmonella* suspension (approximately 8 log CFU/mL) for 120 s. During immersion, the eggs were gently agitated on a rocker to facilitate bacterial attachment. The inoculated eggs were then air-dried at room temperature for 1 h in a biosafety cabinet. A total of 16 eggs were used at each time point across all groups.

### 2.3. Egg Washing and Evaluation of Bactericidal Efficacy Against Three Salmonella Serovars

Following bacterial attachment, eggs were subjected to washing treatments in a water bath using freshly prepared 150 ppm NaOCl. The washing was performed at four different temperatures (35, 40, 45, and 50 °C) for three exposure durations (15, 30, and 45 s). Unwashed inoculated eggs served as controls to determine the initial bacterial load. After washing, eggs were air-dried for 15 min in a biosafety cabinet. To enumerate the surviving *Salmonella*, each egg was placed in a sterile specimen cup containing 45 mL of BPW (BNF Korea Co., Ltd.) and homogenized on a rocker for 2 min. Aliquots (100 μL) of the resulting homogenates, with serial tenfold dilutions prepared as necessary, were plated on xylose lysine deoxycholate (XLD; BNF Korea Co., Ltd.) agar and incubated at 37 °C for 24 h. Typical *Salmonella* colonies, identified as black colonies due to hydrogen sulfide production, were counted the next day, and all experiments were conducted in triplicate to ensure reproducibility.

### 2.4. Statistical Analysis

Statistical analyses were performed using GraphPad Prism version 8.0.2 (GraphPad Software Inc., La Jolla, CA, USA), and data are presented as the mean ± standard deviation of three independent experiments. One-way ANOVA (analysis of variance), followed by Dunnett’s post hoc test, was used to compare each treatment with the unwashed control. Washing temperature and duration were modeled as independent variables, and the dependent variable was the log reduction in CFU for each *Salmonella* serovar on the eggshell surface. An asterisk (*) indicates a statistically significant difference compared with the unwashed control at the same temperature. A *p*-value less than 0.05 was considered statistically significant (* *p* < 0.05, ** *p* < 0.01, *** *p* < 0.001, and **** *p* < 0.0001). The limit of detection for *Salmonella* enumeration was 1.00 log CFU/mL, based on plating 0.1 mL of undiluted homogenate on XLD agar (BNF Korea Co., Ltd.). Values reported as 0.00 log CFU/mL indicate bacterial counts below the limit of detection.

## 3. Results

**Figure 1:**
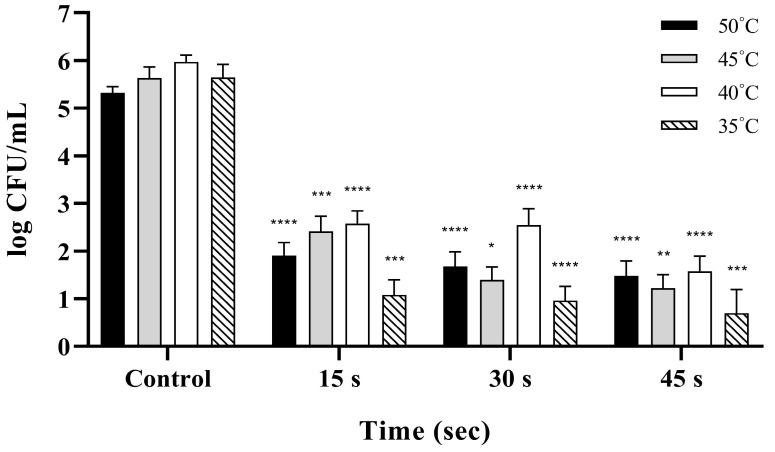
Bactericidal efficacy of 150 ppm NaOCl against *Salmonella* Enteritidis at various washing temperatures and exposure times. The viable bacterial populations are expressed as log CFU/mL, and unwashed eggs were used as the control. Reductions in *S.* Enteritidis were significantly dependent on both washing temperature and time (*p* < 0.001). The highest efficacy was observed at 35 °C for 45 s, achieving a 4.95-log reduction. Asterisks above the bars indicate significant differences compared to the control group (* *p* < 0.05, ** *p* < 0.01, *** *p* < 0.001, and **** *p* < 0.0001). Data are presented as the mean ± standard deviation of three independent replicates.

As shown in Figure 1, reductions in *S.* Enteritidis were significantly influenced by both washing temperature and exposure time (*p* < 0.001). Overall, bacterial loads were reduced by 3–4 log CFU/mL across tested conditions. Notably, exposure time appeared to have a more pronounced effect on efficacy than temperature. Across temperatures, the highest reductions were observed at 35 °C (Figure 1). At this temperature, washing for 30 and 45 s resulted in reductions of 4.83 and 4.95 log, respectively, compared to the unwashed control group. Conversely, the lowest efficacy was recorded at 40 °C, with reductions ranging from 3.31 to 3.87 log depending on the exposure time. At 45 °C, efficacy remained high (4.34–4.82 log), whereas 50 °C produced moderate reductions (3.35–3.95 log) (Supplementary Table S1). Regardless of washing temperature, bacterial inactivation increased with longer exposure times, with 45 s being significantly more effective than 15 s (*p* < 0.001). The optimal washing condition was 35 °C for 45 s, yielding a 4.95 log reduction against *S.* Enteritidis (Supplementary Table S1).

**Figure 2:**
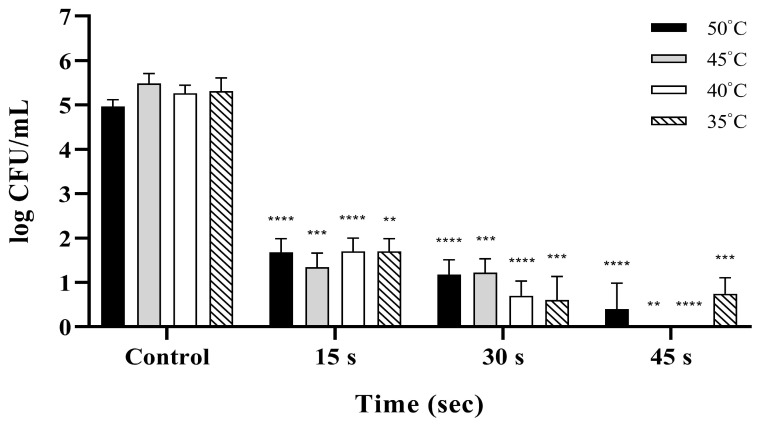
Bactericidal efficacy of 150 ppm NaOCl against *Salmonella* Typhimurium under different washing temperatures and exposure times. The viable bacterial populations are expressed as log CFU/mL, and unwashed eggs were used as the control. Reductions in *S.* Typhimurium were significantly influenced by both washing temperature and duration (*p* < 0.001). The most potent bactericidal effect was achieved at 45 °C for 45 s, resulting in a 5.48 log reduction. Asterisks indicate statistical significance compared to the unwashed control group (** *p* < 0.01, *** *p* < 0.001, and **** *p* < 0.0001). Data are presented as the mean ± standard deviation of three independent replicates.

Similarly to the results for *S.* Enteritidis, reductions in *S.* Typhimurium were significantly dependent on both washing temperature and exposure time (*p* < 0.001; Figure 2). All NaOCl treatment conditions achieved substantial reductions compared to the unwashed group. At 50 °C, longer exposure times increased bactericidal efficacy, yielding up to a 4.16 log reduction (Supplementary Table S2). At 45 °C, the efficacy increased at 30 and 45 s, with reductions greater than those at 50 °C (Supplementary Table S2). A similar trend was observed at 40 °C, where the bactericidal effect was highly time-dependent; prolonged washing at this temperature achieved reductions of up to 5.37 log (Supplementary Table S2). Although washing at 35 °C consistently resulted in reductions greater than 3 log, the efficacy at 45 s was slightly lower than that at 30 s. Among the tested conditions, the strongest bactericidal effect against *S.* Typhimurium was observed at 45 °C for 45 s, with a 5.48 log reduction.

**Figure 3:**
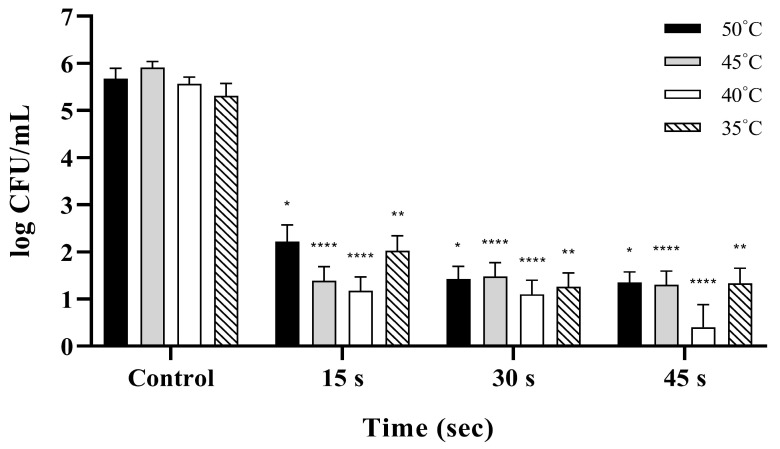
Bactericidal efficacy of 150 ppm NaOCl against *Salmonella* Thompson at various washing temperatures and exposure times. The viable bacterial populations are expressed as log CFU/mL, and unwashed eggs serve as the control group. *S.* Thompson shows significant reductions of 3–5 log units across all tested temperatures (*p* < 0.001). The maximal reduction occurs at 40 °C with a 45 s exposure (5.17-log reduction). Asterisks indicate significant differences from the control group (* *p* < 0.05, ** *p* < 0.01, and **** *p* < 0.0001). Data represent the mean ± standard deviation from three independent replicates.

Bactericidal activity against *S.* Thompson was evident at 150 ppm NaOCl and mirrored the trends observed in the other two serovars, yielding significant reductions of 3–5 log across all temperatures compared to the unwashed control (*p* < 0.001; Figure 3). Bacterial counts declined in a clear time-dependent manner at 50 °C and 40 °C; the most pronounced effect occurred at 40 °C for 45 s, resulting in a 5.17 log reduction (Supplementary Table S3). At 45 °C, all washing conditions achieved reductions exceeding 4 log, with a stronger effect at 15 and 45 s than at 30 s, and the maximum occurred at 45 s. In contrast, efficacy at 35 °C was generally lower than that at other temperatures, and a slight decrease in reduction was noted at 45 s relative to 30 s (Supplementary Table S3). Overall, the highest bactericidal efficacy against *S.* Thompson was achieved at a washing temperature of 40 °C.

## 4. Discussion

In this study, washing eggs with 150 ppm NaOCl significantly reduced bacterial counts on the eggshell surface. The magnitude of reduction depended on washing temperature and exposure time. Bactericidal efficacy also differed among *S.* Enteritidis, *S.* Typhimurium, and *S.* Thompson, indicating serovar-specific responses to NaOCl. These differences likely reflect variation in cell-surface structures or biofilm-forming capacity, which can modulate disinfectant action. Our findings align with previous reports that NaOCl concentration, washing temperature, and exposure duration are key determinants of microbial inactivation on eggshells [14,15,20,21]. Collectively, the data show that washing conditions critically determine the effectiveness in controlling eggshell contamination.

Multiple egg-washing approaches have been used to reduce microbial loads and improve egg safety [14,16,22,23,24,25,26]. In contrast to many earlier reports, we found that 150 ppm NaOCl alone exhibited bactericidal efficacy against *S.* Enteritidis, *S.* Typhimurium, and *S.* Thompson on contaminated eggshells at temperatures of 35 °C or higher. Notably, efficacy was greater at temperatures below 50 °C for all three *Salmonella* serovars, suggesting that excessively high temperatures (e.g., 50 °C) may diminish activity via cuticle damage, chlorine degradation, or increased bacterial tolerance. Consistent with prior studies, increasing temperature does not invariably enhance NaOCl efficacy because excessive heating can reduce chemical stability or available chlorine [27,28]. Moreover, contact time often has a greater impact on inactivation than either concentration or temperature. Here, washing with 150 ppm NaOCl achieved 3–5 log reductions across serovars, depending on temperature and exposure time. Thus, NaOCl can effectively reduce *Salmonella* on eggshells when applied under appropriate washing conditions.

For *S*. Enteritidis, the greatest reduction occurred at 35 °C for 45 s, representing a lower optimal washing temperature than that for *S*. Typhimurium and *S*. Thompson. Chlorine solutions can exhibit higher antimicrobial activity at lower temperatures [24], and NaOCl may inflict more serious oxidative damage at 35 °C than at higher temperatures. Although 150 ppm NaOCl has been reported to reduce *S*. Enteritidis by more than 6.44 log regardless of washing time in controlled liquid systems [15], the reductions observed in the present study were 3–4 log. This difference reflects experimental conditions—including humidity, NaOCl concentration, pH, temperature, contact time, surface characteristics—and variation in initial bacterial loads due to surface irregularities. In particular, the porous and heterogeneous structure of the eggshell surface may limit uniform contact with disinfectants, potentially resulting in lower reductions than those achieved under commercial egg-washing processes where disinfectant solutions are evenly applied [29]. Notably, increasing the washing temperature above 35 °C did not lead to further enhancement of bactericidal efficacy against *S*. Enteritidis under the conditions tested. Overall, *S.* Enteritidis appeared more sensitive to washing temperature, with 35 °C providing the most effective bacterial inactivation under the conditions tested.

According to our results, *S.* Typhimurium showed greater reductions than *S.* Enteritidis at most temperatures except 35 °C, with the maximal effect at 45 °C. This difference may reflect intrinsic physiological traits of *S.* Typhimurium, including a relatively higher resistance to oxidative stress, distinct energy metabolism, and tolerance to chlorine-induced membrane damage [30]. Such characteristics may allow *S*. Typhimurium to better withstand chlorine exposure at lower temperatures, while remaining susceptible under conditions that optimize disinfectant activity. Although the precise mechanism underlying the enhanced efficacy at higher temperatures remains unclear, previous studies suggest that *S*. Typhimurium is capable of activating multiple defense systems in response to chlorine stress, including HOCl (hypochlorous acid)-sensing regulators and oxidative stress-response pathways that facilitate survival under chlorine exposure [31,32,33]. It is therefore possible that washing at moderately elevated temperatures, such as 45 °C, provides conditions that partially overcome these defense mechanisms without substantially compromising NaOCl stability. Taken together, maintaining washing temperatures around 45 °C appears to balance NaOCl stability with bactericidal activity for effective control of *S.* Typhimurium.

The inactivation pattern for *S*. Thompson differed from those of *S*. Enteritidis and *S*. Typhimurium, indicating serovar-specific responses to chlorine-based washing. Our results showed that the strongest bactericidal effect against *S*. Thompson was observed at 40 °C. Notably, the bactericidal response was not strictly time-dependent, as prolonged exposure did not result in proportionally greater inactivation. Similar time-independent behavior has been reported where residual organic matter, eggshell surface irregularities, and microstructural pores reduce the availability of free HOCl over time, thereby limiting sustained disinfectant activity [18,20]. Although *S.* Thompson is not globally dominant it has caused notable foodborne outbreaks in the Republic of Korea [12,34], underscoring its public health relevance in regional contexts. However, in contrast to *S.* Enteritidis and *S.* Typhimurium, data on washing efficacy against *S.* Thompson remain limited. In our study, *S.* Thompson was highly susceptible to 150 ppm NaOCl, showing 3–5 log reductions and robust bactericidal efficacy across the tested conditions. These findings indicate that conventional chlorine-based washing, when applied under appropriate temperature conditions, may be particularly effective against *S*. Thompson contamination. Nevertheless, given the limited number of studies and the observed non–time-dependent inactivation behavior, further research is warranted to define optimal washing conditions for practical application targeting *S.* Thompson.

This study has several limitations. First, the use of a single strain for each *Salmonella* serovar may not fully reflect the genetic and phenotypic diversity of strains encountered in egg production environments. In commercial settings, eggs may be simultaneously exposed to multiple *Salmonella* strains, thereby increasing the potential for cross-contamination and variability in resistance to washing and disinfection processes. Second, the relatively small number of eggs used, along with variability in bacterial attachment due to differences in eggshell surface structure among individual eggs, may have influenced the observed results. Finally, because the disinfection experiments were conducted under laboratory rather than in commercial conditions, the findings may not fully reflect performance under commercial egg-processing environments.

## 5. Conclusions

We demonstrated that 150 ppm NaOCl reduced *S.* Enteritidis, *S.* Typhimurium, and *S.* Thompson on eggshell surfaces by 3–5 log, depending on washing temperature and exposure time. Bactericidal activity varied by serovar; each showed its greatest reduction at a different washing temperature within 35–45 °C. Considering practical egg-processing conditions, our results suggest that washing eggs for at least 30 s with 150 ppm NaOCl solutions maintained at 40–50 °C may enhance microbial control under commercial systems. Under controlled conditions, NaOCl is a reliable egg-washing sanitizer. Standardized protocols specifying NaOCl concentration, temperature, and exposure time could strengthen microbial control in egg-handling facilities. These data support improved egg sanitation practices and a lower risk of *Salmonella* transmission via eggshells. Further studies are necessary to evaluate the long-term effects of NaOCl washing on eggshell integrity and internal egg quality parameters, such as albumen quality and yolk stability following washing treatments. In addition, future research should investigate mixed-strain or cross-contaminated egg models under industrial-scale washing systems to better assess the applicability of laboratory findings to commercial egg-processing environments.

## Data Availability

The data presented in this study are available upon reasonable request to the corresponding author.

## Supplementary Materials

The following supporting information can be downloaded at https://www.mdpi.com/article/10.3390/pathogens15020133/s1, Table S1: Bactericidal effect of 150 ppm NaOCl by wash time and temperature against *Salmonella enterica* subsp. *enterica* serovar Enteritidis; Table S2: Bactericidal effect of 150 ppm NaOCl by wash time and temperature against *Salmonella enterica* subsp. *enterica* serovar Typhimurium; Table S3: Bactericidal effect of 150 ppm NaOCl with wash time and temperature toward *Salmonella enterica* subsp. *enterica* serovar Thompson.

## Abbreviations

The following abbreviations are used in this manuscript:

BPW	Buffered Peptone Water
CFU	Colony Forming Unit
ClO_2_	Chlorine dioxide
HOCl	Hypochlorous acid
MFDS	Ministry of Food and Drug Safety
NaOCl	Sodium Hypochlorite
*S.* Enteritidis	*Salmonella enterica* subsp. *enterica* serovar Enteritidis
*S.* Typhimurium	*Salmonella enterica* subsp. *enterica* serovar Typhimurium
*S.* Thompson	*Salmonella enterica* subsp. *enterica* serovar Thompson
TSA	Tryptic Soy Agar
XLD	Xylose Lysine Deoxycholate
