# Supplementary material for: Bactericidal Efficacy of Sodium Hypochlorite on Eggshells Contaminated with Three Salmonella Serovars
Source: Pathogens. 2026 Jan 26;15(2):133. doi: 10.3390/pathogens15020133 (PMC12943329; doi:10.3390/pathogens15020133)
Supplement: Supplementary file 1 [file pathogens-15-00133-s001.zip › pathogens-4096121-supplementary.pdf]

Supplementary Table S1. Bactericidal effect of 150 ppm NaOCl by wash time and temperature against *Salmonella enterica* subsp. *enterica* serovar Enteritidis.

**Temperature (°C)**	**Time (s)**	**Log CFU/mL (mean ± SD)**	**Log reduction[a]**
**50**	Control	5.32 ± 4.85	
	15	1.90 ± 1.79	3.42
	30	1.58 ± 1.62	3.74
	45	1.47 ± 1.47	3.85
**45**	Control	5.63 ± 5.47	
	15	2.41 ± 2.41	3.22
	30	1.40 ± 1.18	4.23
	45	1.22 ± 1.14	4.41
**40**	Control	5.98 ± 5.58	
	15	2.58 ± 2.45	3.39
	30	2.54 ± 2.54	3.43
	45	1.58 ± 1.56	4.39
**35**	Control	5.64 ± 5.56	
	15	1.08 ± 1.07	4.56
	30	0.96 ± 0.94	4.68
	45	0.70 ± 1.00	4.94

[a]: Log reduction values indicate the decrease in log CFU/mL relative to the unwashed control.

Supplementary Table S2. Bactericidal effect of 150 ppm NaOCl by wash time and temperature against *Salmonella enterica* subsp. *enterica* serovar Typhimurium.

**Temperature (°C)**	**Time (s)**	**Log CFU/mL (mean ± SD)**	**Log reduction[a]**
**50**	Control	4.97 ± 4.56	
	15	1.68 ± 1.64	3.29
	30	1.18 ± 1.17	3.79
	45	0.70 ± 0.82	4.27
**45**	Control	5.49 ± 5.28	
	15	1.34 ± 1.33	4.14
	30	1.22 ± 1.20	4.26
	45	0.00 ± 0.00	5.48
**40**	Control	5.26 ± 4.95	
	15	1.70 ± 1.66	3.57
	30	0.70 ± 0.70	4.57
	45	0.00 ± 0.00	5.26
**35**	Control	5.31 ± 5.27	
	15	1.70 ± 1.64	3.61
	30	0.60 ± 0.96	4.71
	45	0.74 ± 0.84	4.56

[a]: Log reduction values indicate the decrease in log CFU/mL relative to the unwashed control.

Supplementary Table S3. Bactericidal effect of 150 ppm NaOCl with wash time and temperature toward *Salmonella enterica* subsp. *enterica* serovar Thompson.

Temperature (°C)	Time (s)	Log CFU/mL (mean ± SD)	Log reduction[a]
**50**	Control	5.67 ± 5.44	
	15	2.22 ± 2.16	3.45
	30	1.43 ± 1.28	4.24
	45	1.35 ± 1.11	4.32
**45**	Control	5.91 ± 5.40	
	15	1.39 ± 1.35	4.52
	30	1.48 ± 1.43	4.43
	45	1.30 ± 1.24	4.61
**40**	Control	5.57 ± 5.08	
	15	1.23 ± 1.12	4.34
	30	1.10 ± 1.04	4.47
	45	0.40 ± 0.64	5.17
**35**	Control	5.31 ± 5.20	
	15	2.02 ± 2.00	3.29
	30	1.26 ± 1.20	4.05
	45	1.34 ± 1.33	3.97

[a]: Log reduction values indicate the decrease in log CFU/mL relative to the unwashed control.
